# The *eClinical Care Pathway Framework*: a novel structure for creation of online complex clinical care pathways and its application in the management of sexually transmitted infections

**DOI:** 10.1186/s12911-016-0338-8

**Published:** 2016-07-22

**Authors:** Jo Gibbs, Lorna J. Sutcliffe, Voula Gkatzidou, Kate Hone, Richard E. Ashcroft, Emma M. Harding-Esch, Catherine M. Lowndes, S. Tariq Sadiq, Pam Sonnenberg, Claudia S. Estcourt

**Affiliations:** Blizard Institute, Barts and The London School of Medicine & Dentistry, Queen Mary University of London, London, UK; Research Department of Infection and Population Health, University College London, Mortimer Market Centre, off Capper Street, London, UK; School of Information Systems & Computing, Brunel University London, Uxbridge, UK; School of Law, Queen Mary University of London, Mile End Road, London, UK; HIV/STI Department, Public Health England, London, UK; Institute of Infection and Immunity, St George’s, University of London, London, UK

**Keywords:** Online clinical care pathway, Framework, eHealth, Sexual health, Sexually transmitted infections, Chlamydia trachomatis

## Abstract

**Background:**

Despite considerable international eHealth impetus, there is no guidance on the development of online clinical care pathways. Advances in diagnostics now enable self-testing with home diagnosis, to which comprehensive online clinical care could be linked, facilitating completely self-directed, remote care. We describe a new framework for developing complex online clinical care pathways and its application to clinical management of people with genital chlamydia infection, the commonest sexually transmitted infection (STI) in England.

**Methods:**

Using the existing evidence-base, guidelines and examples from contemporary clinical practice, we developed the *e*Clinical Care Pathway Framework, a nine-step iterative process. Step 1: define the aims of the online pathway; Step 2: define the functional units; Step 3: draft the clinical consultation; Step 4: expert review; Step 5: cognitive testing; Step 6: user-centred interface testing; Step 7: specification development; Step 8: software testing, usability testing and further comprehension testing; Step 9: piloting. We then applied the Framework to create a chlamydia online clinical care pathway (Online Chlamydia Pathway).

**Results:**

Use of the Framework elucidated content and structure of the care pathway and identified the need for significant changes in sequences of care (Traditional: history, diagnosis, information versus Online: diagnosis, information, history) and prescribing safety assessment. The Framework met the needs of complex STI management and enabled development of a multi-faceted, fully-automated consultation.

**Conclusion:**

The Framework provides a comprehensive structure on which complex online care pathways such as those needed for STI management, which involve clinical services, public health surveillance functions and third party (sexual partner) management, can be developed to meet national clinical and public health standards. The Online Chlamydia Pathway’s standardised method of collecting data on demographics and sexual behaviour, with potential for interoperability with surveillance systems, could be a powerful tool for public health and clinical management.

## Background

In common with many other countries, UK has a prominent *e*Health agenda which prioritises self-led and remote care [1–3]. Most eHealth to date has focused on monitoring and management of long-term health conditions, such as asthma, diabetes and hypertension, offered as an adjunct to traditional care [4–6]. Typically patients first receive their medical diagnosis from a healthcare professional in a face-to-face consultation, with opportunities to discuss their condition and its management, before digital technologies such as apps for adherence, health promotion and symptom diaries [4–20] are offered.

Analogous to home pregnancy testing, new infectious disease diagnostic technology means that people will be able to self-test for various infections at home and will be able to self-diagnose with a new medical condition remote from medical services. This could have considerable benefits, particularly for people with stigmatizing infections such as sexually transmitted infections (STIs) and HIV, as people report barriers to accessing services and highly value accessibility and convenience of medical care [21–25]. In England, the major burden of STIs occurs in young people [26]. This group has high smartphone and internet usage, with 88 % of 16–24 year olds owning a smartphone, and being rapid adopters of new technology [27].

The development of home STI diagnostics creates potential for developing a completely remote online clinical care pathway, from diagnosis through to management. However, UK national standards for STI management contain multiple facets of care [28] which are far more complex than simply prescribing antibiotics. These include sexual partner management, health promotion, sign-posting to other related services and collection of data for routine public health surveillance purposes, whilst maintaining data security and patient confidentiality [29]. All these elements would need to be incorporated within an online care pathway for National Health Service use.

Currently there is no guidance in England on the development and content of online clinical care pathways (‘structured multidisciplinary plans of anticipated care’ [30]), and, in particular, no guidance specific to sexual health.

Here we describe a new framework for development of complex online clinical care pathways (see Methods) and demonstrate its utility for development of a system for management of genital *Chlamydia trachomatis* infection (see Results), the commonest STI in England [26], using an online automated clinical consultation followed by remote prescribing and sexual partner management.

## Methods

### Development of the framework

**Fig. 1:**
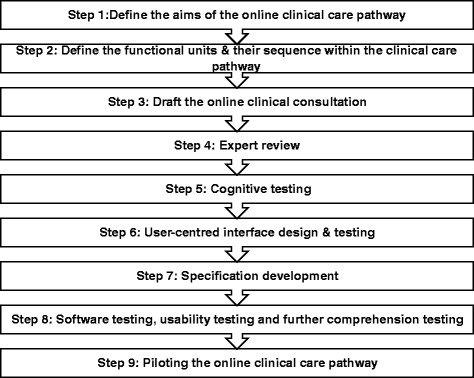
*e*Clinical Care Pathway Framework

Using the existing evidence base, guidelines and examples from contemporary clinical practice, we developed the *e*Clinical Care Pathway Framework. For the Online Chlamydia Pathway, intended to take users, who have tested using a variety of routes, from accessing test result, and therefore diagnosis, through automated medical assessment and on to treatment, we undertook a comprehensive literature review of published and grey literature. We were unable to identify any validated tools or methods to guide the development of a remote online automated clinical care pathway. However, there was information available that could be used to indirectly inform the development of such a pathway. We synthesised our findings to develop the Framework creating a nine-step iterative process (Fig. 1).

This included evidence on Step 1 (aims) [31, 32], Step 2 (pathway sequence) [33], Step 3 (online consultation) [34], Step 4 (expert review) [34–36], Step 5 (cognitive testing) [37–41], Step 6 (interface testing) [39, 42, 43], Step 8 (software and usability testing) [32, 33, 35, 39, 44, 45] and Step 9 (pilot) [31, 35, 41].

Step 1 is to define the aims of the online clinical care pathway. Step 2 requires definition of the functional units by breaking down the clinical care pathway into sections (for example history, examination and investigations (screening or diagnostic tests) based around the traditional aspects of clinical care it contains. It is important to consider whether the sequence needs to be different to traditional care. For example, in a traditional clinical care pathway sequence where a patient comes into clinic, the initial functional unit is the presenting complaint and history of presenting complaint. In an online clinical care pathway this may not be appropriate, particularly if the pathway is linked to, for example, remote self-testing and diagnosis.

Step 3 involves drafting the automated online clinical consultation which is likely to be a key component of most clinical care pathways. This is the “automated medical assessment” which includes history-taking, decision-making and easily extractable and transmissible surveillance data. Composed of clinical and behavioural questions, it is designed to determine whether it is safe and appropriate to proceed with remote management of the condition. Relevant published literature, proformas and protocols in contemporary use, standards and existing services provide a robust evidence-base for the online consultation.

Step 4 involves review of the draft consultation by clinical experts with respect to the content, phrasing and flow of questions and text. Any issues where it is not possible to reach consensus as part of this process can be focussed on as part of Step 5 and Step 6. Immediately following this it is important to ensure that users comprehend and interpret the text and questions correctly. This can be done by cognitively testing [46] (Step 5) the text and questions with a sample of your target population.

Step 6 involves user-centred interface design and testing. For user-centred interface design the question set is translated into a format that works on the chosen screen resolutions with the appropriate response options. It is important to use relevant user interface design guidelines as a basis for this [42]. The purpose of testing is to focus on the users’ views of the interface, how the information is presented and the order of the interactive steps in the user interface.

Step 7 requires conversion of the online clinical consultation into a database specification in order for software engineers to design the system according to one’s needs. This process is described in more detail in the results section below and includes translation of the database specification in to wireframes to specify the user interface to achieve the optimal design output. Once the software engineers have produced an application (app), further expert usability review, software testing, usability testing and comprehension testing can be conducted by a clinical researcher and Human Computer Interface expert (Step 8). Finally, Step 9 involves piloting of the online clinical care pathway with the target population, with further refinement occurring on the basis of findings and evaluation of this.

### Application of the framework to chlamydia

Once the Framework was developed, we then applied it to our exemplar condition, genital chlamydia to produce the Online Chlamydia Pathway. Chlamydia is an ideal candidate infection as it is common, and there is a standard first line antibiotic therapy, Azithromycin 1 g orally [47]. Azithromycin has good tolerability, a low side-effect profile, and allergy and drug interactions are infrequent among the target population [48–53].

This phase involved:

1. Sourcing of national and international clinical standards for sexual history taking, chlamydia management, health promotion, sexual health service provision, consent, good medical practice, and prescribing
2. Collation of protocols (‘a comprehensive set of rigid criteria outlining the management steps for a single clinical condition or aspects of organisation’ [30]) and proformas in use in contemporary sexual health settings
3. Literature search for evidence on provision of STI results and the individual components of the online clinical consultation
4. Collation of sexual health questionnaires and computer assisted structured interviews

## Results and discussion

### Step 1: Aim

The aim of the Online Chlamydia Pathway was to enable people with genital chlamydia to receive their test result online, obtain information about the infection, complete a clinical consultation, and for those for whom it was appropriate to do so, progress to receive a remote prescription of antibiotic treatment in a safe, efficient manner. It also needed to ensure that patients for whom it was not appropriate to be managed in this way were identified and transferred to traditional services in a timely and efficient manner. Further aims of the Online Chlamydia Pathway included partner notification, provision of epidemiological treatment where acceptable and appropriate for sexual partners, a two-week health adviser follow-up phone-call for all patients who accessed the online clinical consultation, and collection of data for national surveillance system purposes.

### Step 2: pathway sequence

**Fig. 2:**
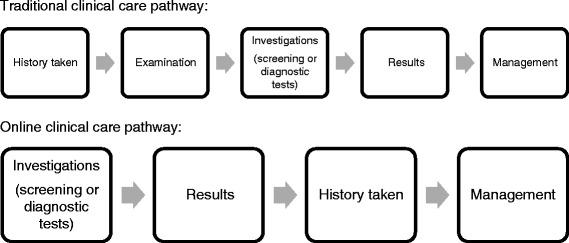
Contrast between the basic sequence of functional units of care in traditional clinical care pathways and online clinical care pathways

Use of the Framework clarified the basic sequence of functional units of care (Fig. 2), which is fundamentally different to traditional care pathways which start with the medical history, followed by examination, investigations, results and management. Our pathway had to accommodate patients entering from different types of chlamydia screening providers, each with different processes for, and content of, medical information gathering and capture at the time of testing, whilst keeping collection of patient identifiable data to a minimum. This meant that there was no patient history available at the point of diagnosis and therefore this had to be taken once the patient was already aware of the results of the investigations.

It is also possible that people may have developed new symptoms in the time between testing and diagnosis, or that they may be more candid in the face of a positive test result, and it was therefore important to establish a history specific to whether it was safe and appropriate to prescribe Azithromycin without face-to-face clinical review. This was a radical departure from traditional pathways and had major implications in terms of the information that needed to be provided and the content, phrasing, logic and order of the questions asked in the clinical consultation.

**Fig. 3:**
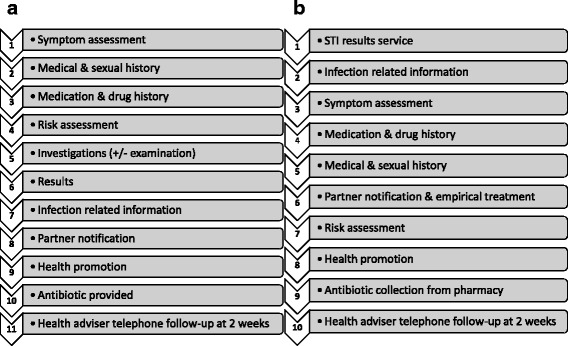
**a** Functional units of a traditional sexual health pathway. **b** Functional units of the Online Chlamydia Pathway

Based on the online pathway sequence, the individual functional units of the pathway were established (Fig. 3a and b). As well as the points made above, the rationale behind the order chosen included reducing the length of the interaction for those whom it was not safe to treat. In this way such patients could transfer to alternative (face-to-face) care as quickly as possible, rather than only finding this out at the end of the consultation.

### Step 3: online consultation

The automated online clinical consultation consisted of functional units one to nine, as shown in Fig. 3b. For each of these units, the objective of the unit and evidence bases for the unit were analysed, with a decision then being made as to the optimal way of developing and implementing that unit. The first functional unit was a results service which required examination of the evidence base [54–67] and current practice to conclude the best way for patients to access their results. Information was collected within the online clinical consultation on basic personal and demographic details (required for both clinical needs and surveillance purposes), taking relevant medical/drug history, symptom assessment, sexual history, risk assessment, and partner notification with epidemiological treatment [38, 43, 48, 51–53, 68–108].

Those patients for whom it was assessed to be safe and appropriate were able to choose a local pharmacy from where they could collect their treatment. A clinical helpline was available to support patients’ medical and psychosocial needs throughout this pathway. All patients who consented [109] were followed up with a phone call from a research health adviser at two weeks. A separate portal was developed to support the research health adviser; this included details and status of all eligible patients, details of information entered onto the system by the results administrator and patient, a screen for documenting conversations via the clinical helpline, treatment outcomes and a structured two week phone follow-up.

### Step 4: expert review

The expert review panel consisted of consultant genitourinary medicine and public health physicians, academics in sexual health, public health, human computer interaction, bioethics and a research health adviser. Feedback from the panel led to amendments to the content and phrasing of the text and questions within the online clinical consultation.

### Step 5: cognitive testing

Beatty and Wallis define cognitive testing as ‘the administration of draft survey questions while collecting additional verbal information about the survey response, which is used to evaluate the quality of the response or to help determine whether the question is generating the information that its author intends’ [110]. Cognitive testing was conducted with members of the public. One of the main findings from this was that people either did not know what azithromycin (a macrolide and first-line treatment for chlamydia [93]) was or confused it with erythromycin. This meant that they were unable to accurately interpret and answer the question relating to allergies. It was therefore necessary to develop a series of questions which allowed patients with no allergies to pass on to the next section whilst ensuring that any patients who were allergic to macrolides came off the online pathway and into clinic.

### Step 6: user-centred interface design and testing

Initial focus groups were conducted by a Human Computer Interaction researcher in university and secondary schools in order to establish user requirements [42]. User interface design principles and guidelines were then used to design the interface to ensure optimal display of questions and response sets, along with facilitating the user journey and flow of interaction. Lab-based user interface testing was conducted with wire-framed prototypes.

### Step 7: specification development

A specification was developed in Microsoft Excel which included text, logic, data item numbering, field names and the export data sheet. This was sent to the software engineers, along with the user interface design wireframes (which specified the look and feel of the interface and clarified the user journey) for development of a demonstration version of the pathway.

### Step 8: software and usability testing

Further software, usability and cognitive testing was then conducted with the demonstration version of the pathway before the system went live. This included the Human Computer Interaction researcher conducting an expert usability review and lab-based testing with members of the public, and repeated testing of the system to ensure that it was accurate, fully functional and coded correctly.

### Step 9: pilot

The Online Chlamydia Pathway has been successfully piloted in an exploratory study involving patients recruited from Sexual Health Clinics and the National Chlamydia Screening Programme in England [111].

## Conclusions

Despite the increasing uptake of eHealth within the NHS, at present there is no guidance available on the development of online clinical care pathways. There are fundamental differences between the approach taken with online compared to traditional clinical care pathways. These include the sequence of the pathway, which questions are asked and how the text is phrased, ensuring that it is safe and appropriate for the patient to be managed online and providing an alternative rapid pathway into care for those patients for whom it is not appropriate.

We developed a new framework for complex online clinical care pathways and adapted it for use for management of genital *Chlamydia trachomatis* infection. The Framework is a useful structure for developing an online clinical care pathway. We have used it here to develop a complex online clinical care pathway for chlamydia which provides the services, data collection, surveillance function and standards a patient would have if attending a traditional service, within an easy and acceptable format. This Online Chlamydia Pathway is compliant with relevant standards and guidance. This is a new method of developing an automated online clinical consultation which is logical, comprehensive and aims to provide users with the ability to choose the management pathway which best suits them, and is most appropriate for them, whilst maintaining a high standard of care. The methodology used could be applied and adapted to a wide range of conditions outside Sexual Health.

We have focused here on a common treatable bacterial STI (*Chlamydia trachomatis*). However, the pathway could be adapted to incorporate testing and managing other STIs, and other services. Indeed, study patients received negative results via the Online Chlamydia Pathway results service for other STI tests they had in clinic. As well as potentially reducing the time to treatment for index patients, and increasing the proportion of patients treated appropriately within the community, this standardised method of collecting data on demographics and sexual behaviour, with easily extractable data and the potential for interoperability with surveillance systems, could be a powerful tool for public health and clinical care. In order to complete this type of process effectively requires effective multi-disciplinary collaboration.

## Abbreviations

NHS, The United Kingdom’s National Health Service; STI, Sexually Transmitted Infection
